# News and Events of the Week

**Published:** 1910-02-19

**Authors:** 


### News and Events of the Week.

Mr. C. H. Golding-Bird has been re-elected to represent the R.C.S. on the Central Midwives Board.

The King has presented the skeleton of his famous Derby winner, Persimmon, to the Natural History Museum, South Kensington.

Dr. William Bramwell Ransom, of Nottingham, who died on December 9, at the age of 48, has left estate valued at £22,386 gross, and £18,849 net.

The King has been graciously pleased to appoint Sir Henry Bell Longhurst, Surgeon-Dentist to his Majesty, to be commander of the Royal Victorian Order.

Mr. Bilton Pollard, F.R.C.S., has been re-elected a member of the Court of Examiners of the R.C.S., and also a member of the surgical section of the Board of Examiners in Dental Surgery.

Four cases of beri-beri are under treatment at the Royal Naval Hospital, Plymouth. The patients were landed from the Brazilian battleship *Minas Geraes*, which arrived in the Sound recently from the Tyne. The men belong to Brazil.

The fourth ordinary meeting of the Royal Statistical Society was held on Tuesday at the Rooms of the Royal Society of Arts, John Street, Adelphi, W.C., when Mr. A. W. Flux, M.A., read a paper on "Urban Vital Statistics in England and Germany."

Mr. Thomas Seccombe, of Granville Mansions, Torquay, who died at the age of 90 in November last, was one of the oldest surgeons on the Retired List of the Royal Navy, and had been present at nearly every engagement during the Kaffir War of 1851; he left estate valued at £3,907 gross, and £3,843 net.

A quarterly meeting of the trustees of the Hunterian Collection was held on February 9 at the Royal College of Surgeons. Dr. A. E. Shipley was elected a trustee in the place of the late Sir Thomas Smith. A committee was appointed to examine into the state of the Hunterian Collection and to report thereon to the trustees at the annual meeting in May.

At an ordinary meeting of the council of the Royal College of Surgeons, held on February 10, a report from the committee appointed to consider the proposals for legislation on anæsthetics was adopted. The committee stated that it had seen the text of a Bill entitled the Anæsthetics Bill, 1909, which was introduced into the House of Commons last June, and had considered the conclusions adopted by the General Medical Council in reference to it. The committee recommended the Council to concur with these except that relating to the administration of anæsthetics by duly qualified dental practitioners. With regard to this conclusion, the committee recommend the omission of the words "during dental operations, or procedures." Having regard to the fact that the services of a medical practitioner with special experience of anæsthetics are often not available, the committee believe that it is no advantage to the public or the profession that dentists be debarred from administering $N_2O$ and the other anæsthetics to be specified.

The late Mr. Henry Evans, of Hove, Brighton, formerly surgeon to the Croydon General Hospital, and resident Obstetric Assistant at Guy's Hospital, has left estate valued at £18,337 gross, and £12,634 net.

An exhibition of apprentices' work, organised by the Apprenticeship and Skilled Employment Association, with the object of promoting a thorough industrial training for boys and girls by apprenticeship and other methods, will be opened by the Lord Mayor on the 24th inst. at the Drapers' Hall, Throgmorton Street.

With a view of demonstrating the advance that has been made in the machinery of the laundry and in the equipment of public baths and other institutions, the Society of Laundry Engineers is organising an exhibition at the Royal Agricultural Hall, London, in April next. This display of laundry machinery and sanitary appliances will include machinery in motion, practical work in progress, demonstrations of new processes, and other departments that should impress the public and educate the laundryman. Conferences on methods of work will be held, and altogether the exhibition is likely to prove an attractive event in the spring.

The question of the appointment of women doctors to resident posts at the Manchester Royal Infirmary was considered last week by the Rochdale Guardians, who are subscribers to the Infirmary. The General Purposes Committee recommended the Board to vote against the appointment of women. An amendment was moved that the Guardians should vote in favour of the appointment of women; 45 per cent. of the patients at the Infirmary were women, and women doctors were well qualified to discharge the duties at the Infirmary. The clerk said the trustees of the Infirmary thought that there were administrative and sentimental reasons against the appointment of women doctors. A Guardian said they had arrived at a time when sentiment should be put aside, and they ought not to stand in the way of the appointment of women doctors on the staff of the Infirmary. He thought that the step would add to the dignity and the efficiency of the institution. The amendment was lost by fifteen votes to nine, and the Guardians will therefore vote against the appointment of women.

With the aid of the Carnegie Trustees medical inspection of school children has been established in Dunfermline. Dr. Bridge, the medical officer, in the annual report, directs attention to two developments: First, the appointment of a school nurse who visits children at their homes, besides inspecting them in the schools; second, the establishment of a school clinic to treat children for whom the proper measures could not otherwise be provided. During the past year Dr. Bridge and his staff have examined 2,130 boys and 2,040 girls. Dr. Bridge also recommends the establishment of a children's labour bureau in connection with the work of medical inspection. "As it is proposed," he says, "to examine all the children three months before they leave school, it would be quite easy to furnish particulars as to physical fitness of any child about to leave school and requiring employment. This report, together with one as to mental attainments supplied by the headmasters, would form the basis of a bureau for the employment of children on leaving school. It would present advantages to the employers of labour as well as to the children."

In his annual report, the Medical Officer of Health for Hampshire states in reference to Aldershot that "it is no exaggeration to say that the vast majority of working-class families let their houses in lodgings. It is right to mention that there is an enormous demand for such lodgings in consequence of the very large number of soldiers married off the strength, and therefore being compelled to provide accommodation for their wives and families in the town. Many such families (each occupying one room only) are to be found in a single dwelling-house, consequently the surroundings generally from such a system are not conducive to good health."

---

The coroner's jury has returned its verdict, writes the *Times* correspondent, in the case in which Dr. Bennett Clarke Hyde filed a suit for slander against the executor of the estate of the late millionaire Colonel Thomas H. Swope. Dr. Hyde alleged that the executor accused him of having caused the death of Colonel Swope and of the latter's nephew. The plaintiff was the medical attendant of Colonel Swope's family and had married one of his nieces. The jury found that Colonel Swope died of strychnine contained in a capsule prescribed by Dr. Hyde; whether with felonious intent the jury was unable to say. He has now been arraigned at Independence, Kansas on a charge of murder, and admitted to bail of £5,000.

---

On Friday, February 25, at 7.30 p.m., a discussion will take place on "Treatment of School Children," under the auspices of the Royal Sanitary Institute at the County Offices, St. Mary's Gate, Derby, Sydney Barwise, M.D., D.P.H., M.O.H., Derbyshire C.C., will open the discussion. The chair will be taken by H. D. Searles-Wood, F.R.I.B.A. (Chairman of the Council of the Institute). On Saturday, February 26, visits will be made (1) to Langley Mill School and Cookery Centre, Sandiacre School, Long Eaton Higher Elementary School and Pupil Teacher Centre, now nearing completion; and (2) to one or two of the newest Council Schools in Derby.

---

At a recent meeting of the Sheffield Guardians the Clerk reported that the audit of the accounts had taken place, and he mentioned that there was a surcharge of £4 5s. 2d. on himself. The disallowance was for expenses of the Medical Officer of the hospital in attending a tuberculosis conference in London, upon which he presented a valuable report. The Auditor took the attitude that though the Board was entitled to send three members and the Clerk to the conference, it was not entitled to send the Medical Officer. Therefore he considered it his plain duty to regard the action as illegal, and also that of the Clerk, "who, in a careless moment, paid the expenses." If he had left it over for a cheque to be signed, it would have been the Chairman and Vice-Chairman who would have been surcharged. Mr. Booker said he proposed to appeal against the surcharge, and he asked the necessary permission, which was granted.

---

A matter of intense interest to scientists and laity alike, writes a Melbourne correspondent, is the proposed visit of 100 members of the British Association for the Advancement of Science to Australia in 1913. No doubt is entertained but that the invitation will be accepted, but before issuing the invitations those who are moving in the matter have approached the Federal Government on the question of expenses. The deputation included Sir John Madden, Chief Justice and Chancellor of the University of Melbourne; Professors Orme Masson, Baldwin Spencer, and Laurie, and Dr. J. W. Barrett, lecturer, of the University; Drs. David Grant and W. Moore, Mr. G. H. Knibbs, Federal Statist; Signor P. Baracchi, head of the Observatory, and Mr. Donald MacKinnon, M.L.A., and received a favourable reply from the Prime Minister. It is estimated that £10,000 will be required for travelling expenses, while State Governments, municipal authorities, and private citizens will be relied upon for local hospitality.

---

It is 153 years ago that the Royal Maternity Charity began its work in London, and the Chairman, Mr. Cornelius Barham, at the annual meeting on February 10, pointed out that this assistance by the charity's trained midwives is one of the means of checking infant mortality and reducing the illness which results from want of proper attention in childbirth. Out of 2,351 cases attended during the past year there were five deaths of mothers and 85 stillbirths. The charity also pays for the services of a number of medical practitioners who go to the assistance of the midwives when needed, and money is badly wanted to pay off the debt with which it is burdened.

---

Hospital humour as related in the institutional papers is generally of a very much watered quality. An exception, however, is to be found in the article "Heard about Hospital," which appears in this month's organ of the Royal Free. We quote the following as fair samples :—

Member of Staff (*getting exasperated*).—"Can't that child be kept from crying, Sister?"

Sister.—"Oh no! Dr. C—r is going over its chest."

Small Boy Patient (*anxiously*).—"Nurse! Nurse! I haven't had my Bier's treat yet!"

District Visitor (*to small child at Alexandra Hospital*).—"Tell me, my little dear, do you know who is your Healer?"

Little Girl (*delighted*).—Oh yes! Mr. Berry's our healer, do you know him?"

(*Visitor is seriously shocked but utterly fails in her attempts to explain away Mr. Berry.*)

Patient.—"I've had an operation under chloroform, myself, Doctor. I had a 'sulphur carcase' removed from my mouth."

---

One of the jokes is a trifle hard on the mere male colleague. First student: "Do you know Mr. S—— W—— was married the week before he came on as resident?" Second student: "Really! Was he as frightened as all that?"

